# Dual therapeutic targeting of MYC and JUNB transcriptional programs for enhanced anti-myeloma activity

**DOI:** 10.1038/s41408-024-01117-4

**Published:** 2024-08-19

**Authors:** Judith Lind, Osman Aksoy, Michaela Prchal-Murphy, Fengjuan Fan, Mariateresa Fulciniti, Dagmar Stoiber, Latifa Bakiri, Erwin F. Wagner, Elisabeth Zwickl-Traxler, Martin Sattler, Karoline Kollmann, Sonia Vallet, Klaus Podar

**Affiliations:** 1 Division of Molecular Oncology and Hematology, Department of Basic and Translational Oncology, Karl Landsteiner University of Health Sciences, Krems an der Donau, Austria; 2 Pharmacology and Toxicology, Department of Biological Sciences and Pathobiology, University of Veterinary Medicine, Vienna, Austria; 3 Institute of Hematology, Union Hospital, Tongji Medical College, Huazhong University of Science and Technology, Wuhan, China; 4 Department of Medical Oncology, Dana-Farber Cancer Institute and Harvard Medical School, Boston, MA USA; 5 Division of Pharmacology, Department of Pharmacology, Physiology and Microbiology, Karl Landsteiner University of Health Sciences, Krems an der Donau, Austria; 6 Genes & Disease Group, Department of Laboratory Medicine, Medical University of Vienna (MUW), Vienna, Austria; 7 Genes & Disease Group, Department of Dermatology, Medical University of Vienna (MUW), Vienna, Austria; 8 Division of Internal Medicine 2, University Hospital Krems, Krems/ Donau, Austria

**Keywords:** Haematological cancer, Pathogenesis

## Abstract

Deregulation of transcription factors (TFs) leading to uncontrolled proliferation of tumor cells within the microenvironment represents a hallmark of cancer. However, the biological and clinical impact of transcriptional interference, particularly in multiple myeloma (MM) cells, remains poorly understood. The present study shows for the first time that MYC and JUNB, two crucial TFs implicated in MM pathogenesis, orchestrate distinct transcriptional programs. Specifically, our data revealed that expression levels of MYC, JUNB, and their respective downstream targets do not correlate and that their global chromatin-binding patterns are not significantly overlapping. Mechanistically, MYC expression was not affected by JUNB knockdown, and conversely, JUNB expression and transcriptional activity were not affected by MYC knockdown. Moreover, suppression of MYC levels in MM cells *via* targeting the master regulator BRD4 by either siRNA-mediated knockdown or treatment with the novel *proteolysis targeting chimera* (PROTAC) MZ-1 overcame bone marrow (BM) stroma cell/IL-6-induced MYC- but not MEK-dependent JUNB-upregulation and transcriptional activity. Consequently, targeting of the two non-overlapping MYC- and JUNB-transcriptoms by MZ-1 in combination with genetic or pharmacological JUNB-targeting approaches synergistically enhanced MM cell death, both in 2D and our novel dynamic 3D models of the BM milieu as well as in murine xenografts. In summary, our data emphasize the opportunity to employ MYC and JUNB dual-targeting treatment strategies in MM as another exciting approach to further improve patient outcomes.

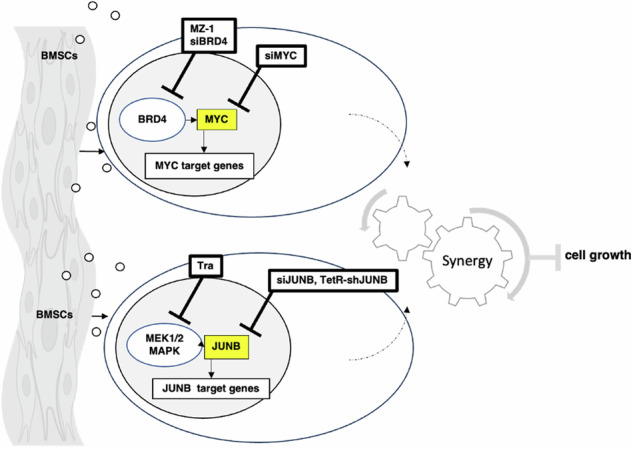

## Introduction

Multiple myeloma (MM) is characterized by clonal expansion of malignant plasma cells within the bone marrow (BM) compartment, monoclonal immunoglobulin in the blood and/or urine, bone lesions, renal compromise, and immunodeficiency. Based on unprecedented advances in our understanding of the pathogenesis of this disease during the last 20 years and the development of derived innovative therapeutics such as Immunomodulatory Drugs (IMiDs), proteasome inhibitors, CD38- and CS1/SLAMF7-targeted monoclonal antibodies, *B cell maturation antigen* (BCMA)-targeted therapies, and the *Exportin-1* (XPO-1) inhibitor selinexor, survival rates in MM patients have increased significantly, approaching a median overall survival (mOS) of 10 years. However, outcomes remain poor for approximately 20% of patients who present with high-risk disease defined by host- or tumor-related factors. The identification of additional novel therapeutic targets and the development of derived novel agents is therefore needed [1–4].

Microenvironment-induced signaling by MEK/MAPK-, PI3K/AKT-, and NFκB-pathways converge and regulate the activity of numerous transcription factors (TFs). Commonly deregulated in MM cells, TFs result in perturbed transcriptomes, which lead to uncontrolled cell proliferation, drug resistance, increased BM angiogenesis, and lytic bone lesions [5, 6].

The basic helix–loop–helix leucine zipper (bHLH-LZ) TF MYC regulates the expression of 10–15% of all genes of the genome and thereby orchestrates cell growth, differentiation, metabolism and death. Following the landmark discovery that MYC translocates to the immunoglobulin loci in human and murine plasmacytoma cells in the early 1980s [7, 8], subsequent studies demonstrated that MYC dysregulation occurs in ∼50% of newly diagnosed MM patients and is associated with decreased progression-free (PFS) and OS. Of note, MYC translocations including IgH (t(8;14)), IgL (t(8;22)), and IgK (t(8;2)), gains and amplifications occur only rarely in monoclonal gammopathy of undetermined significance (MGUS) and smoldering MM (SMM), precursor conditions of MM [6, 9–14]. Supporting a key role of MYC in MM pathogenesis, forced MYC expression in germinal center B cells drives MM progression in Vk*MYC mice [12, 15]. Besides the PI3K/AKT/mTOR pathway, MYC is regulated by members of the *bromodomain and extra-terminal domain* (BET) family of epigenetic readers that facilitate RNA polymerase II (RNA pol II)-mediated transcription. In MM, BET member bromodomain-containing protein 4 (BRD4) binds with high affinity to the super/enhancer region of MYC [16], recruits the positive transcription elongation factor b (P-TEFb) followed by RNA pol II phosphorylation at the site of hyperacetylated chromatin, and ultimately results in transcriptional elongation [17, 18]. Based on its pivotal role in MM pathogenesis, MYC represents an attractive therapeutic target to lower disease burden [19, 20].

Similar to MYC, members of the bZIP activator protein-1 (AP-1) family of TFs play an essential role in a multitude of physiologic processes, but also tumorigenesis. AP-1 TFs are composed of homodimers formed by JUN (CJUN, JUNB, and JUND) proteins as well as heterodimers between JUN and FOS (FOS, FOSB, FOSL1, FOSL2) proteins. AP-1 TF can additionally interact with non-bZIP proteins at gene promoters, including the p65 subunit of NFκB, CBP/p300, and Rb [21, 22]. We previously demonstrated a key role for the AP-1 family member JUNB in MM pathogenesis, showing that MEK/ MAPK- and NFκB-dependent induction of JUNB in MM cells is essential for MM cell proliferation and survival; as well as for the protection against dexamethasone- and bortezomib-induced cell death [23]; and MM BM angiogenesis [24]. As a consequence and similar to MYC, also members of the AP-1 family have evolved as actively pursued therapeutic targets in MM over the past years [25].

While we and others hypothesize that TFs such as MYC and JUNB are excellent targets with a potentially high therapeutic index, they have been traditionally regarded as “undruggable”. Indeed, their active sites are broad, shallow pockets that are difficult to bridge; and small molecules are commonly not able to bind to the smooth surfaces of TFs [26]. However, novel therapeutic strategies are fundamentally changing this paradigm. For example, JQ1, a tert-butyl synthetic precursor of OTX015, is a prototype BET inhibitor (BETi), which competitively binds to the bromodomain and displaces BRD4 from super-enhancers for the MYC oncogene [16, 27–30] thus impeding tumor cell growth in various malignancies, including MM [16, 31–36]. Nevertheless, its reversible binding to BRD proteins and its short half-life cause incomplete transcriptional repression of MYC. Moreover, continued dosing of JQ1 increases the development of drug resistance [37]. To address this problem, PROTeolysis Targeting Chimeric molecule (PROTAC) technology has emerged as maybe *the* most exciting, innovative approach to target TFs by protein degradation [5, 26, 38]. Specifically, PROTACs consist of a ligand for binding to one of more than 600 E3 ubiquitin ligases (most commonly *cereblon* [CRBL] and Von–Hippel–Lindau [VHL]), and the other one (“the warhead”) for binding to a specific protein of interest (POI) as a target for degradation, connected via a linker [39–43]. The spatial proximity allows the formation of a cooperative ternary complex supporting ubiquitination and proteasomal degradation of a specific POI.

Here, we identified for the first time the existence of non-overlapping MYC- and JUNB-regulated transcriptional programs. Moreover, utilizing the investigative BRD4-targeted PROTAC MZ-1 in combination with genetic or pharmacologic JUNB targeting approaches, we emphasize the opportunity to employ MYC and JUNB dual-targeting treatment strategies in MM, as an exciting approach to further improve patient outcome.

## Materials and methods

### Reagents

Recombinant human interleukin-6 (IL-6) (Cat#206-IL/CF) protein was from R&D Systems (Minneapolis, MN, USA); DAPI and doxycycline were from Merck/Sigma Aldrich (Darmstadt, Germany). Antibodies against human MYC (9E10), JUNB (C-11), ERK2 (D-2), and VHL (VHL40) were from Santa Cruz Biotechnology (Dallas, TX, USA); antibodies against PARP (9542S) and cleaved caspase 3 (D175) were from Cell Signaling Technology (Frankfurt am Main, Germany); anti-BRD4 (A301-985A50) was from Bethyl (Montgomery, TX, USA). Trametinib (Cat# HY-10999) was purchased from Med Chem Express (Sollentuna, Sweden), and MZ-1 (Cat# 6154) from Tocris Bioscience (Abingdon, UK).

### Gene dependency mapping

MYC- and JUNB-dependent MM cell vulnerabilities were determined utilizing the CHRONOS algorithm, which leverages the detailed behavior of pooled clustered regularly interspaced short palindromic repeats (CRISPR) experiments in order to improve inference of gene essentiality derived from the *Dependency Map* portal (DepMap Public 23Q2 and 24Q2+Score, Chronos; https://depmap.org/portal). CHRONOS exhibits the lowest copy number and screen quality bias of evaluated methods and models the observed sgRNA depletions across screens and time points to determine the effect of gene knockout on cell growth rate, along with other parameters [44, 45].

### Protein-protein interaction network of MYC and JUNB

Using a minimum required interaction score with high confidence (0.7000), the online database of the search tool for the retrieval of interacting genes/proteins (STRING) Version 12.0 (https://string-db.org) was utilized to investigate potential protein-protein interaction (PPI) networks of MYC or JUNB protein, respectively, on the basis of Homo sapiens. Active interaction sources include text mining, experiments databases, and neighborhoods [46].

### Gene correlation analyses

Coefficient correlation plots were generated using the SRplot online analysis and plotting module, which was written using R/Python language [47].

### ChIP-seq data analyses

For MYC chromatin immunoprecipitation sequencing (ChIP)-seq, raw data were downloaded from *Gene Expression Omnibus* (GEO) (www.ncbi.nlm.nih.gov/geo) with the accession ID GSE36354 [48]. Sample GSM894108 (MM1S_MYC_DMSO) was ChIP against MYC in MM.1S cells, and sample GSM894110 (MM1S_WCE_DMSO_1) served as a control. The resulting fastq files of clean reads were aligned to human genome hg38_94 using BWA with BWA-mem algorithm to generate bam files (BWA, version 0.7.15-r1140, http://bio-bwa.sourceforge.net/). Peaks were called using MACS (version 2.1.1.20160309) with *p* < 0.001 and visualized by Integrative Genomics Viewer (version 2.8.9, Broad Institute). JUNB ChIP-seq data were previously obtained [24], and analyzed in parallel with MYC ChIP-seq data. For occupancy comparison, the overlap of bound regions (at least one base pair in common) of JUNB and MYC were identified using Bedtools intersect (version 2.26.0, http://quinlanlab.org/software.html). Venn diagram and heatmap displaying the overlap between binding peaks of JUNB and MYC were prepared with the VennDiagram package and pheatmap package in R, respectively (http://www.R-project.org/).

### Cell culture and transient transfection

MM tumor cell lines MM.1S, RPMI8226, DOX-40, U266, NCI H929, OPM-1, KMS-12BM, as well as the KM-105 stroma cell line, were purchased from ATCC (Manassas, VA, USA) and DSMZ (Braunschweig, Germany). Human MM cell lines, as well as primary MM cells and BMSCs, were cultured in RPMI-1640 GlutaMAX™ medium supplemented with 10% heat-inactivated fetal bovine serum and 1% penicillin/streptomycin (Gibco, Thermo Fisher Scientific Inc., Waltham, MA, USA). All experiments were conducted using cells that have undergone less than 20 passages after thawing. All cell lines were authenticated through short tandem repeat testing; and tested regularly for the absence of mycoplasma. Tumor cell-stroma cell co-culture experiments were performed as previously described [23].

In some experiments, MM cell line cells were transiently transfected with small interfering RNA (siRNA) SMARTpools for MYC, BRD4, and JUNB, or a non-targeting control (mock) siRNA (Horizon/Dharmacon™ RNA Technologies, Lafayette, CO, USA) using the Lonza™ Nucleofactor™ Transfection 2b device together with the Cell Line Nucleofector Kit V Solution (Lonza Biosciences, Basel, Switzerland).

### Retroviral/lentiviral constructs and transduction

For lentivirus-mediated inducible shRNA knockdown, MM cells were transiently or stably transduced with a pRSIT17-U6Tet-sh-CMV-TetRep-2A-TagGFP2-2A-Puro lentivirus, in which the JUNB-specific shRNA sequence (shJUNB #1 from RNAi Consortium shRNA Libraries [clone ID: TRCN0000014943], TRC/Broad Institute, Cambridge, MA, USA) or scrambled control sequence was inserted resulting in TetR-shJUNB and TetR-SCR (Cellecta/Biocat, Heidelberg, Germany), as previously described [23, 24].

### Quantitative reverse transcription-polymerase chain reaction (RT-qPCR)

Cells were treated and RNA was extracted using the Qiagen RNA isolation kit (Qiagen, Netherlands) according to the manufacturer’s protocol; and RNA concentrations were quantified using the TECAN Infinite 200 PRO system (Tecan, Grödig, Austria). mRNA was used for cDNA synthesis using the iScript cDNA synthesis kit (Bio-Rad Laboratories, Vienna, Austria), and cDNA samples were then analyzed by quantitative reverse qPCR using the Bio-Rad-SSO Advanced Universal SYBR Green Supermix (Biorad, Vienna, Austria), as previously described [23]. Primer pairs used are shown in Supplemental Table 1.

### Cell lysis and western blot analysis

Whole-cell lysates were prepared in RIPA lysis buffer (150 mM NaCl, 10 mM Tris pH 7.2, 0.1% SDS, 1% Triton X-100, 1% deoxycholate and 5 mM EDTA) supplied with the Halt Protease and Phosphatase Inhibitor Cocktail (Pierce, Darmstadt, Germany). Western blot analysis was performed as previously described [23]. Specifically, cell lysates (30–100 μg per lane) were separated by sodium dodecyl sulfate–polyacrylamide gel electrophoresis prior to electrophoretic transfer onto nitrocellulose membranes (Bio-Rad Laboratories, Vienna, Austria). The blots were probed with the respective antibodies prior to incubation with horseradish peroxidase-conjugated secondary antibodies and exposure to the enhanced chemiluminescence substrate.

### MYC and AP-1 reporter gene assays

TetR- shJUNB/MM.1S cells were transiently transfected with HMB-Luc or 3× AP-1 reporter or pGL2-basic vector, together with pRL-CMV Renilla luciferase reporter as an internal control. After the indicated treatment, cell lysates were prepared and luciferase activity was measured using a Dual-Luciferase Reporter Assay System (Promega, Madison, Wisconsin, USA) in a plate reader (SpectraMaxi3x, Molecular Devices, LLC, San Jose, CA, USA) according to the manufacturer’s instructions. The firefly luciferase relative light units (RLU) were normalized to Renilla luciferase RLU. The 3× AP-1 reporter was a gift from Alexander Dent (Indiana University School of Medicine, Indianapolis, IN, USA; Addgene plasmid 40342; http://n2t.net/addgene:40342). HBM-Luc was a gift from Linda Penn (Addgene plasmid 35155; http://n2t.net/addgene:35155).

### Cell cycle analysis

Propidium iodide (Sigma Aldrich, Germany)-stained cells were analyzed on a Cytoflex Beckman Coulter cytometer, and the percentage of cells in G1, S, and G2/M phases was determined using the FlowJo™ v10 Software (Ashland, OR, USA).

### Cell growth assays

The anti-tumor effect of siJUNB, siBRD4, MZ-1, trametinib alone or in indicated combinations on MM cell line and primary MM cell growth was determined by measuring MTS formazan formation (abcam, Cambridge, UK), according to the manufacturer’s instructions on a TECAN Infinite 200 PRO Tecan (Tecan, Gröding Austria).

### 3D model and confocal microscopy

To generate the three-dimensional (3D) model, Qtracker™ 625 (Invitrogen, Waltham, USA)-stained KM-105 stroma cells were pre-seeded overnight onto poly-ε-caprolactone scaffolds (PCLS) (3D BioTek®, Bridgewater, NJ, USA). TetR-shJUNB/MM.1S cells were added on the next day in the presence or absence of doxycycline (1 µg/ml). The loaded scaffolds were subsequently transferred into the 3D-RCCS™ bioreactor (Synthecon Inc., Houston, USA) with or without MZ-1 and co-cultured for 72 h in RPMI-1640 media with 2% fetal bovine serum (FBS). Scaffolds were then fixed in 4% paraformaldehyde (PFA), stained with DAPI or cleaved caspase-3, and imaged using confocal microscopy Leica TCS SP8 X (Vienna, Austria) [49].

### In vivo studies

Animal experiments were conducted at the Institute of Pharmacology and Toxicology, Department of Biological Sciences and Pathobiology of the University of Veterinary Medicine Vienna. NOD.Cg-Prkdc^scid^-Il2rgtm1^Wjl/^SzJ (NOD scid gamma NSG™) mice, which neither express the Prkdc nor the X-linked Il2rg gene, were bred in-house. All animals were housed under specific pathogen-free conditions according to recommendations of the European Laboratory Animal Science Association. All experiments were performed with age-matched 8- to 12-week-old animals with a mean body weight of 20–25 g. We used the statistical software GINGER Tool (https://clinicalbiometrics.shinyapps.io/GINGER/) or R to calculate the sample size for our study. The group sizes were determined to achieve a statistical power of 90%. The required group sizes were reviewed and approved as part of the animal experiment applications. Animals used in the study were blinded. Randomization was not done. In brief, mice were subcutaneously inoculated with 5 × 10^6^ TetR-shJUNB/MM.1S together with 1.5 × 10^6^ human-derived BMSCs and VitroGel Hydrogel Matrix (The Well Bioscience) in 100 μL of RPMI-1640 medium into the flanks. After the randomization of mice and 2 days after inoculation, viral expression was induced by the addition of doxycycline to the drinking water. In addition, a set of mice was treated with the MEK1/2 inhibitor trametinib (1 mg/kg) p.o. instead of doxycycline either alone or in combination with MZ-1 (5 mg/kg) i.p. Once tumors became palpable under the skin, they were measured using caliper measurements every 48–72 h. When the first tumor/s reached a maximum diameter of 1.5 cm, animals were euthanized by cervical dislocation. Statistical significance of different tumor sizes was assessed by one-way ANOVA followed by Tukey’s Multiple Comparison Test using GraphPad Prism.

### Statistical analysis

The Pearson correlation coefficient was used to measure the linear relationship between MYC, JUNB, and their respective target gene mRNA expression levels among RRMM and NDMM patients in the GSE6477 dataset as well as RRMM patients in the GSE31161, the GSE2113, and the GSE13591 datasets by the SRplot online analysis and plotting module which was written by using the R/Python language [47] as well as IBM SPSS for Windows v 26 (https://www.ibm.com/uk-en/analytics/spss-statistics-software; SPSS). Drug combination responses were calculated based on the highest single agent (HSA) reference model using SynergyFinder 3.0 (https://synergyfinder.fimm.fi/) [50, 51].

## Results

### Non-overlapping MYC- and JUNB-transcriptional programs in MM cells

**Fig. 1:**
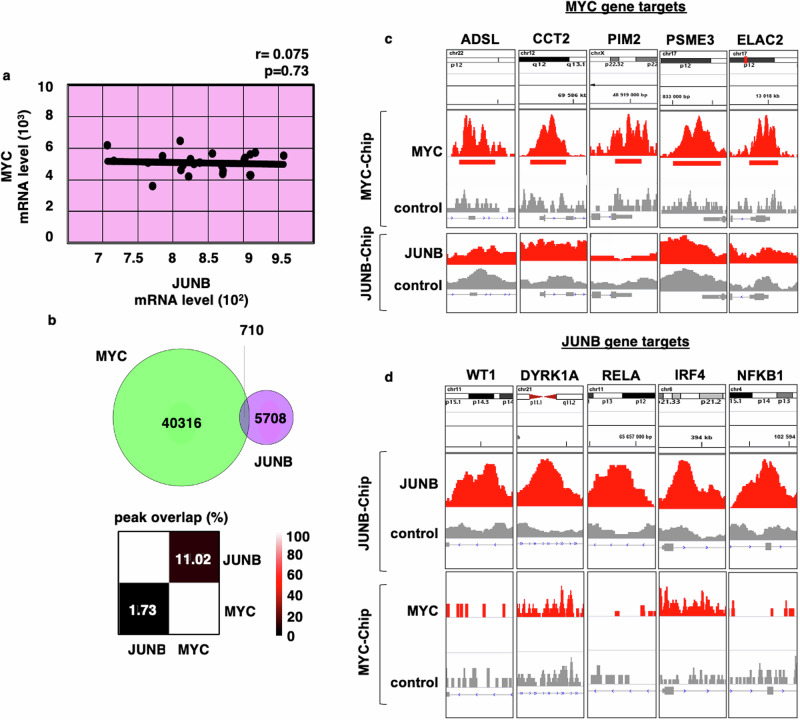
Correlation and ChIP-seq analyses indicate the existence of non-overlapping MYC- and JUNB-transcriptional programs in MM cells. **a** Lack of correlation of expression levels of transcription factors MYC and JUNB. Scatterplot of correlation analysis for MYC and JUNB in the CCLE dataset. The Pearson correlation coefficient was calculated to evaluate the correlation between MYC and JUNB. The minimal level of significance was *p* < 0.05. **b** Marginal overlaps in MYC and JUNB binding peaks. Venn diagram displaying the overlap of MYC with JUNB peaks (upper panel). Heatmap displaying the percentage of overlap between peaks of MYC and JUNB (lower panel). **c** Representative ChIP-seq tracks for MYC (upper track) and JUNB (lower track) at the loci of MYC target genes ADSL, CCT2, PIM2, PSME3, and ELAC2, visualized by genome browser *Integrative Genomics Viewer* (IGV). **d** Representative ChIP-seq tracks for JUNB (upper track) and MYC (lower track) at the loci of JUNB target genes WT1, DYRK1A, RELA, IRF4, and NFKB1, visualized by genome browser IGV. Peaks considered in this analysis were called significant using MACS with *p* < 0.001.

While our own and other previous data have demonstrated that MYC and JUNB play crucial roles in MM pathophysiology, the biological and clinical impact of their transcriptional interference is unknown. Our analyses revealed a lack of correlation between MYC and JUNB mRNA expression levels in the CCLE (Fig. 1a) and the patient-derived GSE6477, GSE31116, GSE2113, GSE13591 datasets (Supplemental Fig. 1a–f), as well as in the large longitudinal, prospective CoMMpass dataset (release IA19-https://research.mmrf.org) (Supplemental Fig. 1g). Furthermore, predicted direct (physical) and indirect (functional) protein-protein interactions (PPIs) utilizing STRING analysis Version 12.0 (https://string-db.org) showed a lack of common PPIs of MYC and JUNB networks (Supplemental Fig. 2a, b). In line with these results, MYC and JUNB expression levels significantly correlated with their respective downstream targets, such as ADSL and PIM2 for MYC and IRF4 and NFKB for JUNB, but not vice versa, in the GSE6477 subset of RRMM patients (Supplemental Fig. 3a, b). Based on the CHRONOS score, an algorithm for inferring gene knockout fitness effects based on an explicit model of cell proliferation dynamics after CRISPR gene knockout [45], several of the MYC and JUNB gene targets are preferentially essential for MM cells, including lineage-defining TFs such as PIM2 for MYC, and IRF4 for JUNB (Supplemental Fig. 3c) as well as regulatory proteins within the endoplasmatic reticulum, the nucleus, the lysosome, the mitochondria, and the Golgi apparatus (Supplemental Fig. 3d). In agreement with our dataset analyses, ChIP-seq analyses in MM.1S cells revealed only marginal overlap between MYC and JUNB binding peaks (merely 1.73% of MYC binding peaks overlapped with those of JUNB, and only 11.02% of JUNB binding peaks overlapped with those of MYC) (Fig. 1b), further emphasizing the existence of exclusive MYC and JUNB transcriptomes in MM cells. For example, binding peaks identified for MYC target genes ADSL and PIM2 in the MYC ChIP-seq analysis were not found in the JUNB ChIP-seq analysis (Fig. 1c). Conversely, peaks observed for JUNB target genes IRF4, NFKB1, and RELA in the JUNB ChIP-seq analysis were not detected in the MYC ChIP-seq analysis (Fig. 1d).

Taken together, these results demonstrate the existence of two non-overlapping MYC- and JUNB- transcriptional programs in MM cells.

### Silencing of MYC but not JUNB abrogates IL-6-induced MYC mRNA and protein levels

**Fig. 2:**
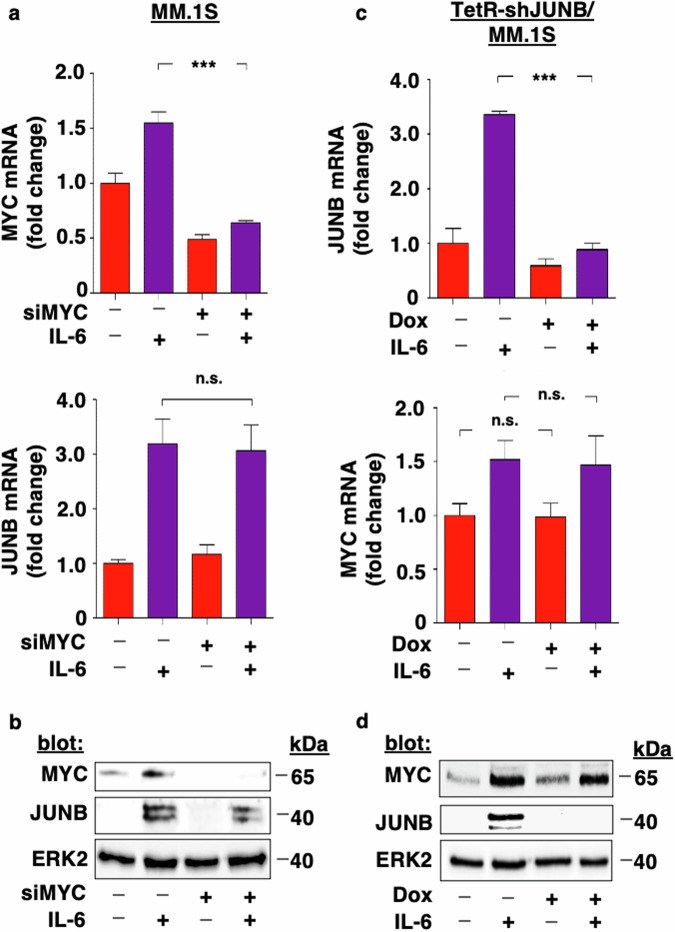
Silencing of MYC but not JUNB abrogates IL-6-induced MYC mRNA and protein levels. **a**, **b** siRNA-mediated silencing of MYC inhibits IL-6-induced upregulation of MYC but not JUNB mRNA and protein levels. MM.1S cells were transiently transfected with siMYC versus control and stimulated with IL-6 (25 ng/ml). After 24 h, MYC and JUNB mRNA (**a**) or protein levels (**b**) were determined using RT-qPCR or immunoblotting with indicated antibodies, respectively. ERK2 served as a loading control. **c**, **d** Doxycycline-induced inhibition of JUNB inhibits IL-6-induced upregulation of JUNB but not MYC mRNA and protein levels. IL-6-stimulated versus control TetR-shJUNB/ MM.1S cells were treated with doxycycline or left untreated. After 24 h, MYC and JUNB mRNA (**c**) or protein levels (**d**) were determined using RT-qPCR or immunoblotting with indicated antibodies, respectively. ERK2 served as a loading control. Data in **a** and **c** represent mean ± SD for triplicate samples of three independent experiments. ***p < 0.0001; n.s. not significant, Dox doxycycline.

Factors secreted upon BMSC: tumor cell contact, such as IL-6 in particular, play a pivotal role in MM proliferation, survival, and drug resistance. IL-6 upregulates both MYC and JUNB protein levels in MM cells [6, 10–13, 23–25, 52]. In order to verify the lack of functional interdependence between MYC and JUNB, we next assessed the impact of siRNA-mediated silencing of MYC or JUNB, respectively, on IL-6-induced upregulation of these TFs in MM cells. siMYC abrogated IL-6-induced upregulation of MYC (upper panel), but not JUNB (lower panel) mRNA (Fig. 2a) and protein levels (Fig. 2b). In contrast, doxycycline abrogated IL6-induced upregulation of JUNB (upper panel), but not MYC (lower panel) mRNA (Fig. 2c) and protein levels (Fig. 2d) in TetR-shJUNB/ MM.1S cells.

Taken together, these results further support the independence of MYC and JUNB transcriptional programs in MM cells.

### Pharmacological inhibition of MYC with MZ-1 abrogates BMSC- and IL-6-induced MYC but not JUNB mRNA and protein levels, as well as transcriptional activity

BRD4 is an epigenetic key regulator of MYC expression in MM cell lines and primary cells and an attractive target for indirect MYC inhibition [16–20]. MZ-1 is the first, cell-penetrant BET-PROTAC composed of the BRD4- binding moiety JQ1, which is tethered to a non-peptic ligand of the E3 ubiquitin ligase VHL via a PEG linker [16, 53–56].

Of note, in comparison to JQ1, MZ-1 is more stable, event-driven, and demonstrates rapid, prolonged, and strong inhibitory activity at sub-stoichiometric doses, even in JQ1-resistant tumor cells, with significantly less toxicity [57]. Moreover, VHL (Supplemental Fig. 4a) and BRD4 (Supplemental Fig. 4b) are highly expressed across all malignant lymphoid cell lines, including MM cells (Supplemental Fig. 4a–d), and do neither carry any VHL nor BRD4 hotspot or loss of function mutation (Supplemental Fig. 4e–h). High VHL (Supplemental Fig. 4i) and BRD4 (Supplemental Fig. 4j) expression levels were also found in patient MM cells derived from the CoMMpass dataset (release IA19-https://research.mmrf.org). Only one patient carried the missense VHL variant C.515 C > A. Six patients carried chr19:g mutations (*patient 1*: chr19:g.4907810_15319431inv, chr19:g.14844770_15330124del, chr19:g.9964719_15269557dup, chr19:g.14844846_15329984inv, and chr19:g.15070112_15319137inv; *patient 2*: chr19:g.15331047_15389178del; *patient 3*: chr19:g.15331562_15376685del; *patient 4*: chr19:g.15286854_17375326dup; *patient 5:* c.3866_3868del-AGC; *patient 6*: chr19:g.15009218_15320756del) and one a gained BRD4 variant C.3256 C > T mutation. Moreover, expression levels of VHL and BRD4 did not significantly differ across MM disease stages (Supplemental Fig. 4k, l). In contrast to an increasing number of chromosomal aberrations for MYC and increasing expression levels for JUNB [14, 23], no significant changes were observed for VHL and BRD4 across samples derived from normal donors, as well as MGUS, SMM, and MM patients (Supplemental Fig. 4m, n). We argue that these features of both VHL and BRD4 support the therapeutic potential of VHL-recruiting BRD4-targeted PROTACs in MM cells.

**Fig. 3:**
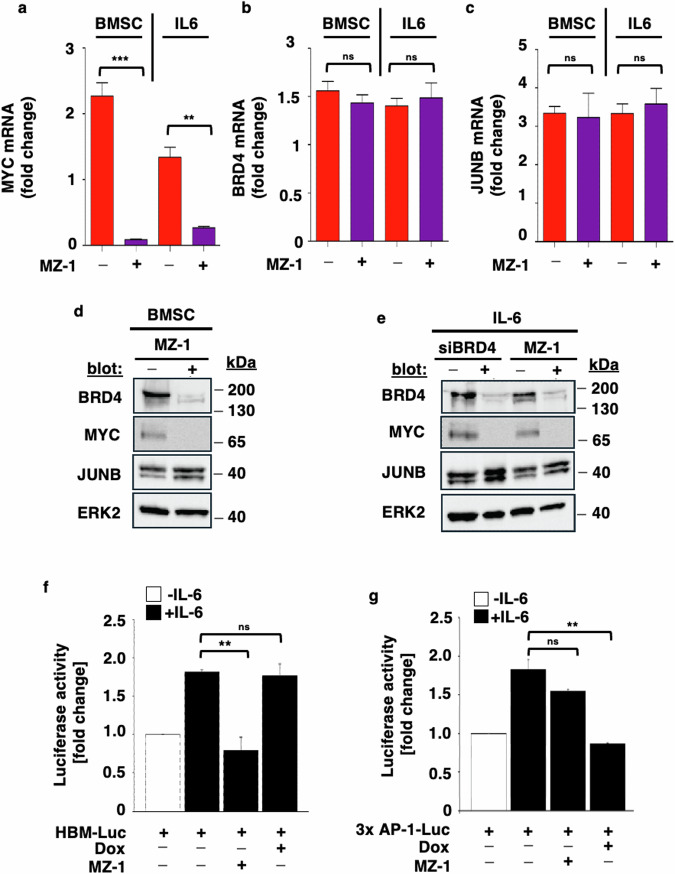
Pharmacological inhibition of MYC with MZ-1 abrogates BMSC- and IL-6-induced MYC but not JUNB mRNA and protein levels, as well as transcriptional activity. **a**–**c** MZ-1-mediated silencing of MYC inhibits BMSC- and IL-6-induced upregulation of MYC, but not BRD4 or JUNB mRNA levels. MM.1S cells co-cultured with BMSCs or stimulated with IL-6 (25 ng/ml) were treated with MZ-1 (100 nM) versus control. After 24 h, MYC, BRD4, and JUNB mRNA were determined using RT-qPCR. Data represent mean ± SD for triplicate samples of three independent experiments. ns non-significant; ^***^ *p* = 0,0004; ^**^ *p* = 0,002. **d** MZ-1 downregulates BRD4 and MYC, but not JUNB protein levels in MM cell: stroma cell co-cultures. After 24 h, lysates were immunoblotted with antibodies against BRD4, MYC, and JUNB. ERK2 served as a loading control. **e** Similar to siBRD4, MZ-1 downregulates MYC protein levels. MM.1S cells were transiently transfected with siBRD4 or treated with MZ-1 versus control and stimulated with IL-6 (25 ng/ml). After 24 h, lysates were immunoblotted with antibodies against BRD4, MYC, and JUNB. ERK2 served as a loading control. **f** Inhibition of IL-6-induced HBM-Luc activity by MZ-1 but not doxycycline. TetR- shJUNB/ MM.1S cells treated with or without MZ-1 or doxycycline were transiently transfected with the HBM-luc reporter together with the pRL-CMV Renilla luciferase vector. Then, the cells were treated with IL-6 or left untreated. Luciferase activity was measured by a dual-luciferase reporter assay. **g** Inhibition of IL-6-induced AP-1 activity by JUNB knockdown but not MZ-1. TetR- shJUNB/MM.1S cells treated with or without doxycycline were transiently transfected with the 3× AP-1 reporter together with the pRL-CMV Renilla luciferase vector. Then, the cells were treated with IL-6 or left untreated. At the indicated time points, luciferase activity was measured by dual-luciferase reporter assay. Data in **f** and **g** represent the fold change of luciferase activity relative to control cells shown as mean ± SD from three independent experiments.

While MZ-1 decreased MYC mRNA levels (Fig. 3a), it had no effect on BRD4 (Fig. 3b) or JUNB (Fig. 3c) mRNA levels in tumor cells stimulated either by adhesion to BMSCs or by IL-6. In agreement with these data, MZ-1 decreased BMSC-induced BRD4 and MYC, but not JUNB protein levels (Fig. 3d). Similar to siRNA- mediated knockdown of BRD4, MZ-1 inhibited IL-6- induced protein levels of BRD4 and MYC, but not of JUNB (Fig. 3e). VHL levels remained unaffected by MZ-1 over a time course of up to 36 h (data not shown). Furthermore, MZ-1 inhibited IL-6-induced MYC- but not AP-1/JUNB-transcriptional activity in TetR-shJUNB/MM.1S cells (Fig. 3f). In contrast, doxycycline inhibited IL-6-induced AP-1/JUNB- but not MYC- transcriptional activity in TetR-shJUNB/ MM.1S cells (Fig. 3g).

Taken together, similar to MYC silencing also pharmacological PROTAC-mediated BRD4 degradation abrogates BMSC- and IL-6-induced MYC but not JUNB mRNA and protein levels as well as its transcriptional activity.

### Dual targeting of MYC and JUNB enhances ex vivo and in vivo anti-myeloma activity

Functionally, MZ-1-reduced cell viability in all (but the resistant RPMI8226 [58]) MM cell line cells investigated (Supplemental Fig. 5a) was associated with an increase of tumor cells in the G0/G1 phase, and a decrease in the S and G2/M phases (Supplemental Fig. 5b). Importantly, MZ-1 also inhibited proliferation of primary MM cells derived from 9 patients (Supplemental Fig. 5c) but had no noticeable effect on mononuclear cells derived from 3 healthy donors (Supplemental Fig. 5d). Since our panel of tumor cells showed different response rates to MZ-1, with RPMI8226 cells being resistant and NCI-H929 cells being most sensitive, we next sought to identify genetic signatures, which are able to predict the response to this treatment in MM cells. A previous study utilizing genome-scale CRISPR gene editing has revealed decreased MZ-1 activity in MM cells treated with sgRNAs against genes including CUL2, VHL, TCEB2, TCEB1, RBX1, UBE2R2, and LZTR1 [59]. In support of these data, our own results demonstrate low expression of these genes in the MZ-1- resistant RPMI8226 cell line versus high expression in the MZ-1-sensitive NCI-H929 and MM.1S cell line cells (Supplemental Fig. 5e). Based on these findings we next generated a multiple linear regression analysis-based mathematical scoring model [*y* = *b* + m1×(P1)+m2×(P2)+m3×(P3)], where [*y*] is the MZ-1- induced IC50 after 24 hours, and (P1-3) are mRNA levels derived from the CCLE dataset of MM cell lines. A least-squares-fit of this equation was used to obtain the constant factors b and m1–3. The predictive ability of the resulting equation [IC50 = 8577 − (1048 × LZTR1) − (1092 × CUL2) + (167 × MYC)] was confirmed by plotting experimental and calculated IC50 of MZ-1 in a total of six MM cell lines (Supplemental Fig. 5f). Applying the equation to calculate the IC50 of MZ-1 also in all other CCLE-MM cell lines indicated high MZ-1 sensitivity (IC50 < 500 nM) in 11% and MZ-1 resistance (IC50 > 2000 nM) in 11% of tumor cells, with the majority of cells (53%) being responsive (Supplemental Fig. 5g). Furthermore, the calculated IC50 of MZ-1 in publicly available MM GSE datasets GSE2658 and GSE2113 indicated high MZ-1 sensitivity in around 30% of patient samples.

**Fig. 4:**
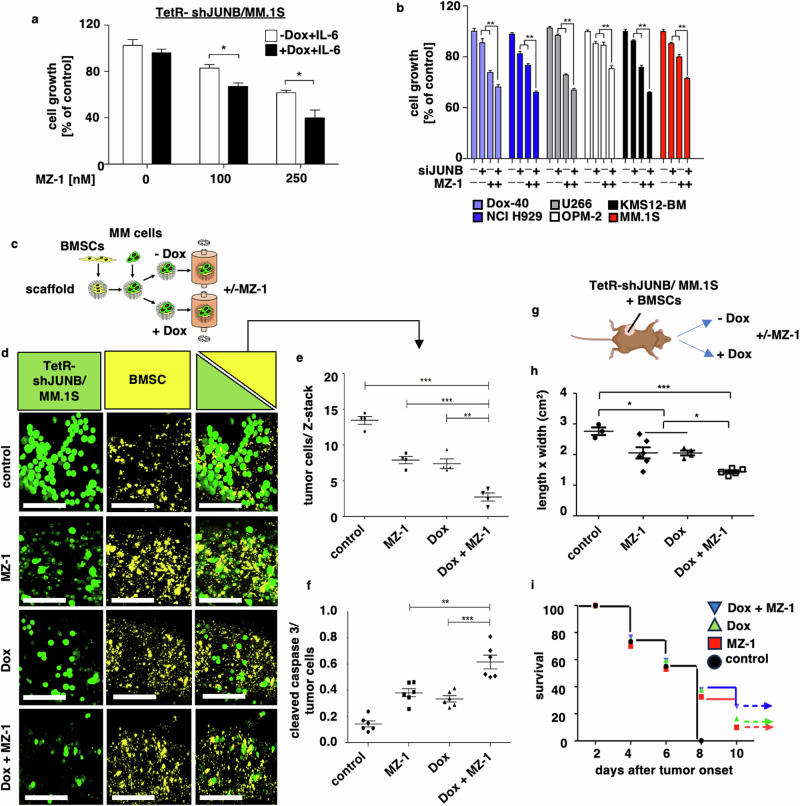
Dual targeting of MYC and JUNB enhances ex vivo and in vivo anti-myeloma activity. **a** MZ-1 increases anti-MM activity of doxycycline-induced knockdown of JUNB. TetR-shJUNB/ MM.1S cells stimulated with IL-6 (25 ng/ml) were treated with doxycycline alone or in combination with MZ-1 for 24 h. Cell growth was measured by fluorescent induction (CyQUANT™). Data represent mean ± SD for triplicate samples of three independent experiments. **b** MZ-1 augments cell death in IL-6-stimulated, siJUNB-treated MM cell lines. MM cell line cells stimulated with IL-6 (25 ng/ml) were transiently transfected with siJUNB versus siControl and treated with MZ-1 versus untreated control for 24 h. Cell growth was determined by an MTS assay. * *p* < 0.01 as compared to control. **p < 0.001; n.s. not significant, Dox doxycycline. **c**–**f** MZ-1 and doxycycline-induced knockdown of JUNB results in synergistic inhibition of MM growth in a dynamic 3D model of the MM BM microenvironment. **c** Dynamic 3D model of the MM BM microenvironment. Qtracker™ 625-stained KM-105 stroma cells were pre-seeded overnight onto poly-ε-caprolactone scaffolds (PCLS). TetR-shJUNB/MM.1S cells were then added in the presence or absence of doxycycline, transferred into the 3D-RCCS™ bioreactor with or without MZ-1, and co-cultured for 72 h in RPMI-1640 media with 2% FBS. **d** Representative Z-stack confocal images of GFP+ TetR-shJUNB/MM.1S cells (green) and Qtracker™ 625-stained KM-105 stroma cells (yellow) cultured alone or together. Scale bars = 100 μm. **e** Quantification of GFP+ TetR-shJUNB/MM.1S cells in Z-stack confocal images of the 3D cultures. **f** Quantification of cleaved caspase-3 TetR-shJUNB/MM.1 S cells in Z-stack confocal images of the 3D cultures. In **e**, **f**, image processing, and analyses were performed with FiJi ImageJ. **g**–**i** MZ-1- and doxycycline-induced knockdown of JUNB results in synergistic inhibition of MM growth in the NSG™ xenograft model of MM. **g** Immunodeficient NSG™ mice were injected subcutaneously with TetR-shJUNB/ MM.1S together with human-derived BMSCs and Matrigel. They were then fed with or without doxycycline in their drinking water and treated with or without MZ-1 (5 mg/kg) i.p. (5×/week). The cartoon was created with BioRender.com. **h** Tumor sizes of TetR-shJUNB/ MM.1S xenografts. **i** Survival curves of mice carrying TetR-shJUNB/ MM.1S xenografts. * *p* < 0.01, ** *p* < 0.001, *** *p* < 0.0001.

Based on the above data, we investigated next, whether dual inhibition of MYC and JUNB is cooperative compared to single inhibition of these TFs. Our results demonstrate enhanced inhibition of tumor cell growth upon combinatorial use of MZ-1 and doxycycline in IL-6-stimulated TetR-shJUNB/MM.1S cells (Fig. 4a). Similarly, MZ-1 also enhanced inhibition of tumor cell growth upon siRNA-mediated JUNB knockdown in various MM cell lines (Fig. 4b). Moreover, in our novel ex vivo dynamic 3D-co-culture model, which closely recreates functional tumor cell: stroma cell interactions (Fig. 4c), MZ-1 significantly augmented doxycycline-induced inhibition of tumor cell proliferation (Fig. 4d, e) and apoptosis (Fig. 4f) of TetR-shJUNB/MM.1S cells. Finally, immune-compromised NSG mice were injected subcutaneously with TetR-shJUNB/ MM.1S cells together with human-derived BMSCs and Matrigel, and treated with either doxycycline, MZ-1 or a combination of both (Fig. 4g). Our results showed that compared to the control group, MZ-1 or doxycycline alone decreased the tumor size (Fig. 4h) and consequently prolonged mouse survival (Fig. 4i); and that dual targeting of MYC and JUNB by MZ-1 and doxycycline, respectively, significantly enhanced their anti-MM activity (Fig. 4g–i).

Taken together, in agreement with our discovery of two independent MYC and JUNB transcriptomes, our results demonstrate that combined *versus* single inhibition of MYC and JUNB significantly enhances the inhibition of MM cell growth.

### Dual targeting of MYC and MEK1/2 enhances anti-myeloma activity

**Fig. 5:**
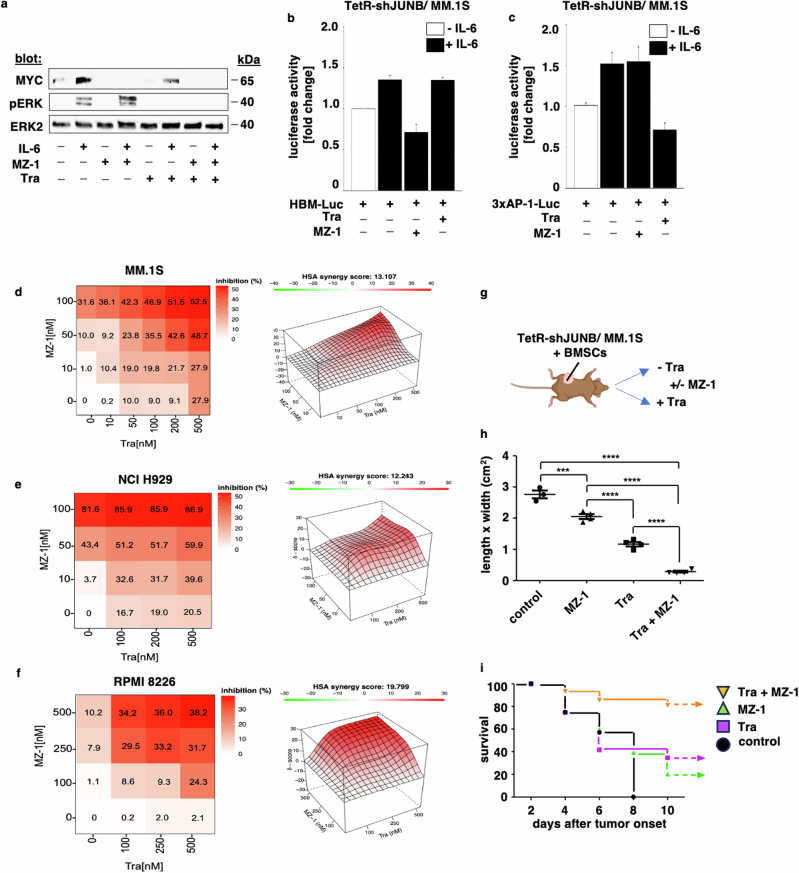
Dual targeting of MYC and MEK1/2 enhances anti-myeloma activity. **a** In contrast to the MEK inhibitor trametinib, MZ-1 does not inhibit the phosphorylation of ERK2. MM.1S cells were treated with MZ-1 or trametinib alone or in combination and stimulated with IL-6 (25 ng/ml). After 24 h, lysates were immunoblotted with antibodies against MYC, pERK2. ERK2 served as a loading control. **b** MZ-1 but not trametinib inhibits IL-6-induced HBM-Luc activity. IL-6 (25 ng/ml)-stimulated TetR- shJUNB/ MM.1S cells treated with MZ-1 or trametinib *versus* control were transiently transfected with the HBM-luc reporter together with the pRL-CMV Renilla luciferase vector. Then, cells were treated with IL-6 or left untreated. Luciferase activity was measured by a dual-luciferase reporter assay. **c** Trametinib but not MZ-1 inhibits IL-6-induced AP-1 activity. TetR- shJUNB/ MM.1S cells treated with or without trametinib were transiently transfected with the 3× AP-1 reporter together with the pRL-CMV Renilla luciferase vector. Then, cells were treated with IL-6 or left untreated. Luciferase activity was measured by dual-luciferase reporter assay. Data in **b** and **c** represent the fold change of luciferase activity relative to control cells shown as mean ± SD from three independent experiments. **p < 0.001 as compared with control. **d**–**g** Synergistic increase of MZ-1-induced anti-MM activity by trametinib. Heat maps represent the percentage of inhibition achieved by MZ-1 and trametinib separately and in combination in MM.1 S (**d**), NCI-H929 (**e**), and RPMI8226 (**f**) MM cell lines. Cells were incubated with increasing concentrations of MZ-1 and trametinib for 24 h and inhibition of cell growth was determined by MTS assay (left panels). Synergy scores were determined by the HSA method (right panels). An HSA synergy score less than −10 is considered to indicate antagonistic, a synergy score between −10 and +10 additive, and a synergy score >10 synergistic anti-MM effects. The percentage of cell growth inhibition is depicted in a colorimetric scale from red (high) to green (low) normalized to DMSO (control). HSA [51], highest single agent. Data represent mean ± SD for triplicate samples of three independent experiments. **g**–**i** MZ-1-mediated degradation of MYC and trametinib-induced JUNB inhibition result in synergistic anti-MM growth in an NSG™ xenograft MM model. **g** Immunodeficient NSG™ mice were injected subcutaneously with TetR-shJUNB/ MM.1S together with human-derived BMSCs and Matrigel. They were then fed with or without trametinib (1 mg/kg) in their drinking water and treated with or without MZ-1 (5 mg/kg) i.p. (5×/week). **h** Tumor sizes of TetR-shJUNB/MM.1S xenografts. **i** Survival curves of mice carrying TetR-shJUNB/ MM.1S xenografts. Tra trametinib.

IL-6-triggered JUNB upregulation is dependent on ERK2 phosphorylation [23]. We next sought to determine the impact of MZ-1 on ERK2 activation. Our results demonstrate that in contrast to the orally available, allosteric MEK inhibitor trametinib, treatment with MZ-1 did not reduce ERK2 phosphorylation. In contrast, trametinib inhibited ERK2 phosphorylation but did not modify MYC protein levels. Consequently, a combination of MZ-1 with trametinib abrogated both MYC protein levels as well as ERK2 phosphorylation levels (Fig. 5a). Moreover, MZ-1 inhibited IL-6- induced MYC (Fig. 5b) but not AP-1/JUNB (Fig. 5c) transactivation activity in TetR-shJUNB/MM.1S cells. In contrast, trametinib inhibited IL-6-induced AP-1/JUNB but not MYC (Fig. 5b) transactivation activity in these tumor cells. Functionally, strong synergistic anti-MM activity was observed upon combinatorial use of MZ-1 with trametinib in MM cell lines MM.1S, NCI H929 (Fig. 5d, e), as well as in MZ-1- resistant [58] RPMI 8226 cells (Fig. 5f). Importantly, trametinib also significantly synergized with MZ-1 in our MM NSG xenograft model (Fig. 5g–i).

Taken together, similar to genetic JUNB-targeting approaches, the in vitro and in vivo anti-MM activity of MZ-1 was also significantly enhanced by its combination with the MEK1/2 inhibitor trametinib.

## Discussion

TFs represent the convergence points of intrinsic and extrinsic signaling pathways and account for approximately 10% of all genes in the human genome, thus representing the single largest family of human proteins; among them, ~20% are oncogenes including MYC and JUNB [5]. Indeed, aberrant transcriptomes are responsible for disease initiation, uncontrolled proliferation, survival, and drug resistance of MM cells within the BM microenvironment. However, ongoing worldwide efforts are needed to further improve our understanding of the pathophysiologic contribution of transcriptomes, their complexity and grade of interference in MM biology, with the ultimate aim to uncover therapeutic opportunities and to guide derived treatment decisions.

The present study investigated for the first time the functional relationship between MYC and JUNB, two essential TFs implicated in MM disease. Utilizing dataset analyses as well as genetic approaches, our results revealed that MYC- and JUNB-induced transcriptomes in MM cells are independent. Consequently, we next explored the functional consequences of single *versus* combined inhibition of MYC and JUNB in MM cells. We co-treated MM cells with the VHL-recruiting BRD4-PROTAC MZ-1 and genetic approaches directly, as well as a pharmacologic approach to indirectly target JUNB *via* blockade of MEK1/2. Indeed, dual targeting of MYC and JUNB by MZ-1 and siJUNB- or shJUNB-mediated knockdown, or the orally available MEK1/2- inhibitor trametinib, demonstrated synergistic anti-MM activity in various MM cell line cells, including MZ-1-resistant RPMI8226 cells [58]. Of interest, our early results indicate that JUNB inhibition upregulates CUL2 expression in RPMI8226 cells thereby potentially sensitizing them to MZ-1. Ongoing studies seek to verify these data.

The therapeutic exploration of MYC and AP-1 TFs such as JUNB has just begun [20, 25]. Doubtlessly, approaches to directly or indirectly target TFs currently emerge among the most promising novel anti-MM strategies with a potentially high therapeutic index. Continuing basic and translational research on TFs is fundamental to once more improve MM treatment strategies and, thereby, patient outcomes in the near future. Specifically, continuing efforts to improve TF inhibitors, protein degraders in particular, aim [1]: at overcoming the “hook effect” (formation of an ineffective binary instead of a ternary complex at a PROTAC concentration above a certain threshold) [2]; at improving their tissue/cell permeability and better assess their distribution, metabolism, and excretion [3]; at optimizing their design (including enhancement of their binding affinity, reduction of their molecular weight, and optimization of their linker design) [4]; at further minimizing their on-target off-tumor and off-target toxicity; and [5] at identifying rationally derive combination partners [60].

In summary, our results demonstrate for the first time that MYC- and JUNB-regulated transcriptional programs are non-overlapping in MM and provide the rationale for dual MYC: JUNB targeting treatment strategies in MM. Moreover, our data strongly support further efforts to develop new TF inhibitors and optimize protein degraders as an exciting new class of therapeutics that are likely to become a potent new therapeutic armamentarium for MM.

### Supplementary information

Supplemental data

## Data Availability

The datasets generated during and/or analyzed during the current study were derived from the *Cancer Cell Line Encyclopedia* (CCLE) portal, an online encyclopedia of a compilation of gene expression, chromosomal copy number, and massive parallel sequencing data from 947 human cancer cell lines (https://portals.broadinstitute.org/ccle/data). RNA-seq data derived from MM patients for MYC, JUNB, and downstream target genes analyzed in this study are publicly available under *Gene Expression Omnibus* (GEO)2R accession codes *Genomic Spatial Event* (GSE)6477, GSE31116 (other treatment relapse), GSE31116 (TT2 relapse), GSE13591, and GSE2113 (https://www.ncbi.nlm.nih.gov/gds). Use of the *Multiple Myeloma Research Foundation* (MMRF) CoMMpass data (release IA19-https://research.mmrf.org) on MM patient samples was approved by the data access use committee and downloaded from dbGaP. Data for non-MM patient samples were derived from *The Cancer Genome Atlas* (TCGA) Research Network (https://portal.gdc.cancer.gov/, http://cancergenome.nih.gov/). For MYC *Chromatin immunoprecipitation sequencing* (ChIP)-seq, raw data were downloaded from *Gene Expression Omnibus* (GEO) (www.ncbi.nlm.nih.gov/geo) with the accession ID GSE36354 [48]. JUNB ChIP-seq data were previously obtained [24].
