# Supplementary material for: Dual therapeutic targeting of MYC and JUNB transcriptional programs for enhanced anti-myeloma activity
Source: Blood Cancer J. 2024 Aug 19;14(1):138. doi: 10.1038/s41408-024-01117-4 (PMC11333473; doi:10.1038/s41408-024-01117-4)
Supplement: Supplementary file 1 — Supplemental data [file 41408_2024_1117_MOESM1_ESM.pdf]

Supplemental data

Supplementary Table 1: Primers used for quantitative real-time PCR.

Gene	Forward	Reverse
**MYC**	5'-CACCAGCAGCGACTCTGA-3'	5'-GATCCAGACTCTGACCTT-3'
**JUNB**	5'-ATGGAACAGCCCTTCTACCACG-3'	5'-AGGCTCGGTTTCAGGAGTTTG-3'
**BRD4**	5'-GAGCTACCCACAGAAGAAACC-3'	5'-GAGTCGATGCTTGAGTTGTGTT-3'
**GAPDH**	5'-GAAGGTGAAGGTCGGAGT-3'	5'-CATGGGTGGAATCATATTGGAA-3'

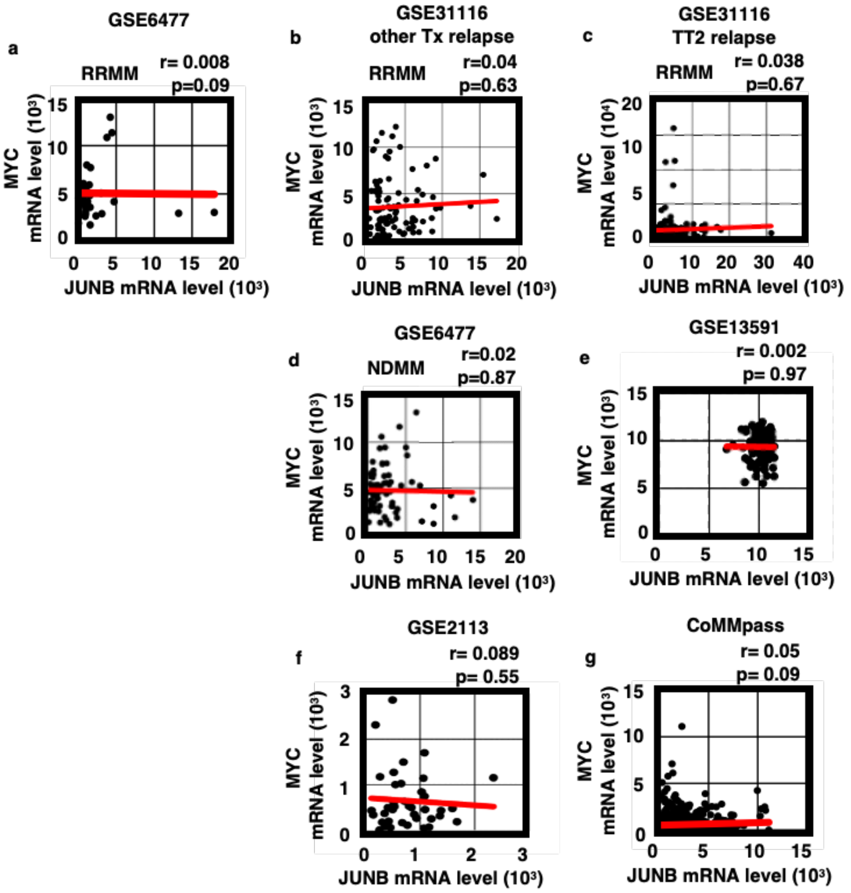

Supplemental Figure 1. Lack of correlation of expression levels of transcription factors MYC and JUNB in patient MM datasets. Scatterplots of correlation analysis for MYC and JUNB in the GSE6477 (relapsed/ refractory subpopulation) (a), GSE31116 (other Tx relapse) (b), GSE31116 (TT2 relapse) (c), GSE6477 (newly diagnosed subpopulation) (d), GSE13591 (e), GSE2113 (f), and the CoMMpass (g) datasets. The Pearson correlation coefficient was calculated to evaluate the correlation between MYC and JUNB. The minimal level of significance was $p < 0.05$.

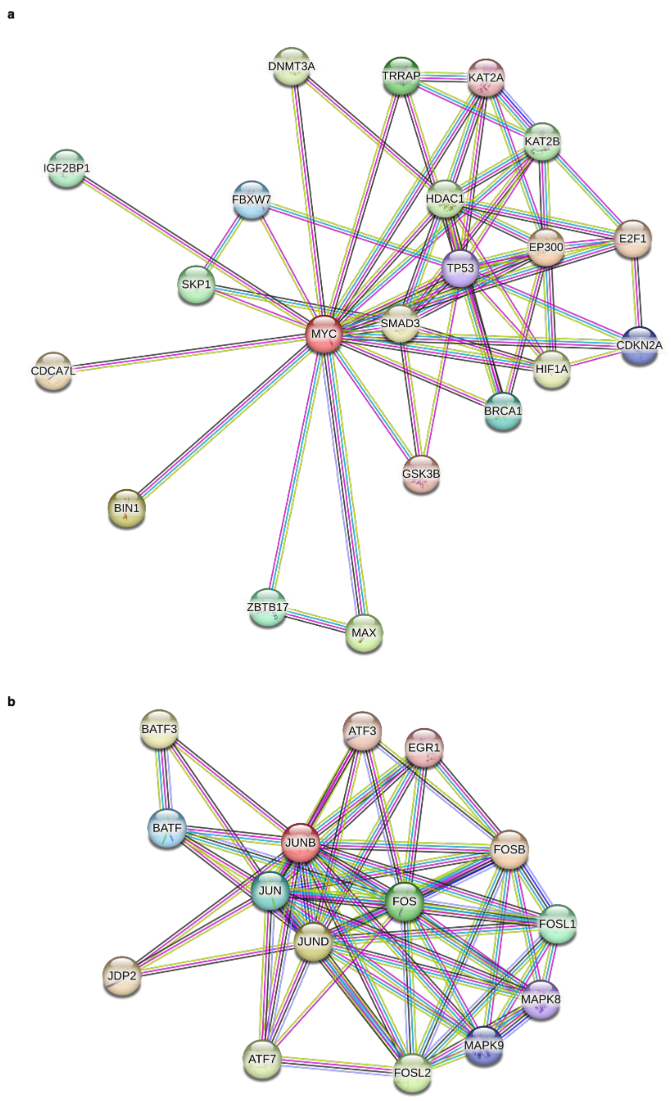

Supplemental Figure 2. Protein-protein interactions (PPIs) of MYC and JUNB predicted by STRING Version 12.0 (https://string-db.org/). (a) Protein-protein interactions (PPIs) of MYC predicted by STRING. (b) PPIs of JUNB predicted by STRING. The edges indicate that the proteins are part of functional or physical protein associations. Coloured nodes for MYC (n = 11) and JUNB (n= 11) represent proteins and the first shell of interactors (average node degree for MYC = 4.91 and for JUNB = 8.55). Number of edges represent specific and meaningful protein-protein associations for MYC (n = 27, of an expected number of edges of n = 15 PPI enrichment p-value 0.00465) and JUNB (n = 47, of expected n = 11, PPI enrichment p-value 6.66e-16) (i.e., proteins jointly contribute to a shared function). The maximum number of interactors shown is 10. Coloured lines between the proteins indicate the various types of interaction evidence. Red line - indicates the presence of fusion evidenc; Green line - neighbourhood evidence; Blue line – co-occurrence evidence; Purple line - experimental evidence; Yellow line – text-mining evidence; Light blue line - database evidence; Black line – co-expression evidence.

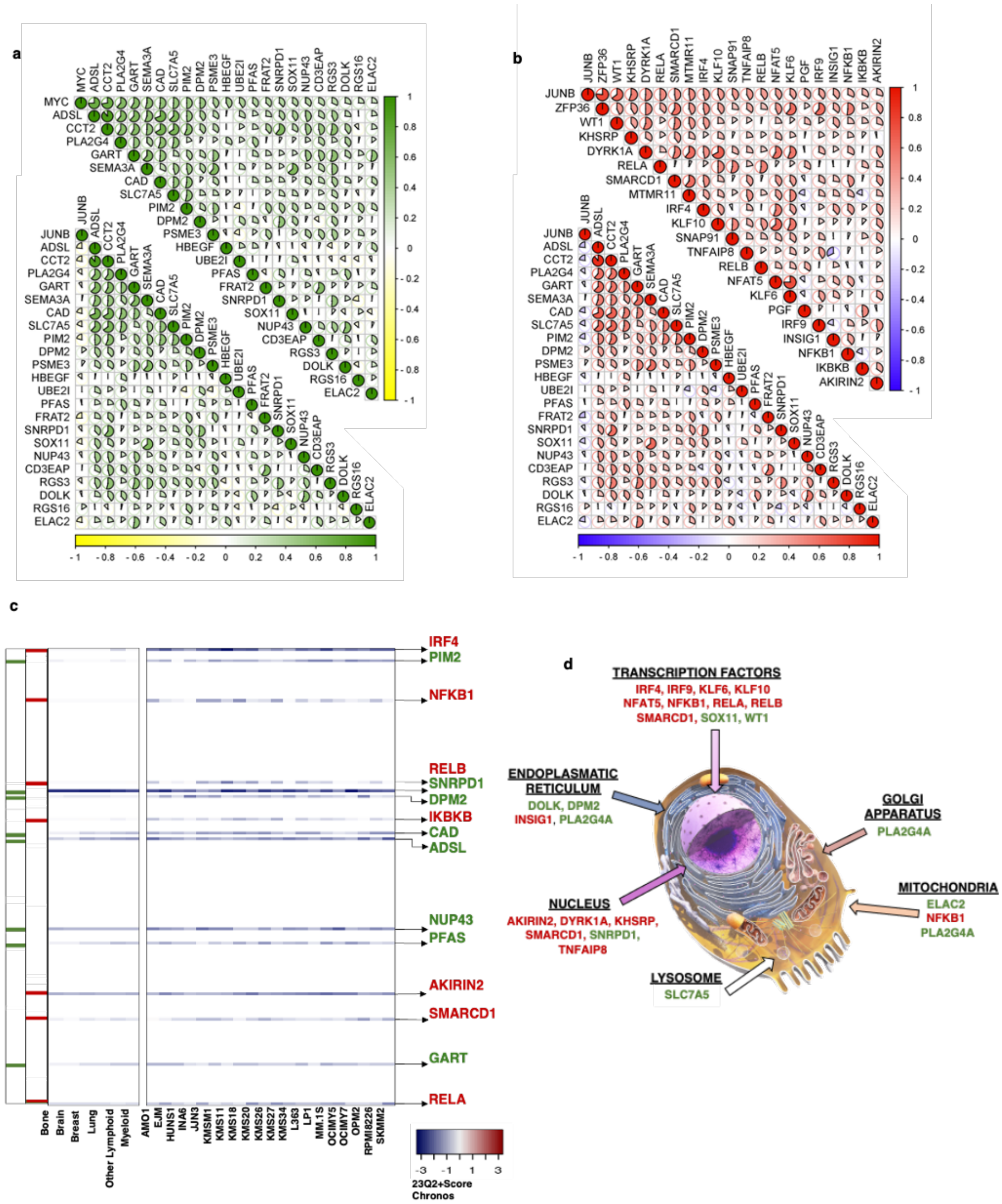

Supplemental Figure 3. Correlation analyses indicate the existence of non-overlapping MYC- and JUNB- transcriptional programs in MM cells.

(a, b) Lack of correlation between putative MYC and JUNB gene targets in the patient- derived GSE6477 dataset. Putative MYC gene targets (in green) (a) and JUNB gene targets (in red) (b) are depicted with the pie method and were generated using the SRplot online analysis and plotting module

which was written by using the R/Python language. mRNA expression in the patient- derived GSE6477 dataset was determined using log2 normalized expression values of probe sets 202431_s_at (MYC), 202144_s_at (ADSL), 201946_s_at (CCT2), 210145_at (PLA2G4), 212378_at (GART), 206805_at (SEMA3A), 202715_at (CAD), 201195_s_at (SLC7A5), 204269_at (PIM2), 209391_at (DPM2), 209852_x_at (PSME3), 203821_at (HBEGF), 208760_at (UBE2I), 213302_at (PFAS), 209864_at (FRAT2), 202690_s_at (SNRPD1), 204914_s_at (SOX11), 219007_at (NUP43), 205264_at (CD3EAP), 220300_at (RGS3), 204488_at (DOLK), 209324_s_at (RGS16), 201766_at (ELAC2) for putative MYC target genes; and 201473_at (JUNB), 201531_at (zfp36), 216953_s_at (WT1), 204372_s_at (KHSRP), 209033_s_at (DYRK1A), 201783_s_at (RELA), 209518_at (SMARCD1), 213511_s_at (MTMR11), 216987_at (IRF4), 202393_s_at (KLF10), 204953_at (SNAP91), 210260_s_at (TNFAIP8), 205205_at (RELB), 215092_s_at (NFAT5), 208961_s_at (KLF6), 209652_s_at (PGF), 203882_at (IRF9), 201625_s_at (INSIG1), 209239_at (NFKB1), 209341_s_at (IKBKB), 213810_s_at (AKIRIN2) for putative JUNB target genes. (c) MYC and JUNB target genes are preferentially essential for MM fitness. Heat maps depict the 150 top CHRONOS gene effect scores of MYC- and JUNB- target genes in non-MM and MM cell lines. Data were accessed through the DepMap portal (DepMap Public 23Q2+Score, Chronos). (d) Schematic representation of MYC- (green) and JUNB- (red) gene targets and their subcellular localization. MYC gene targets, green; JUNB gene targets, red.

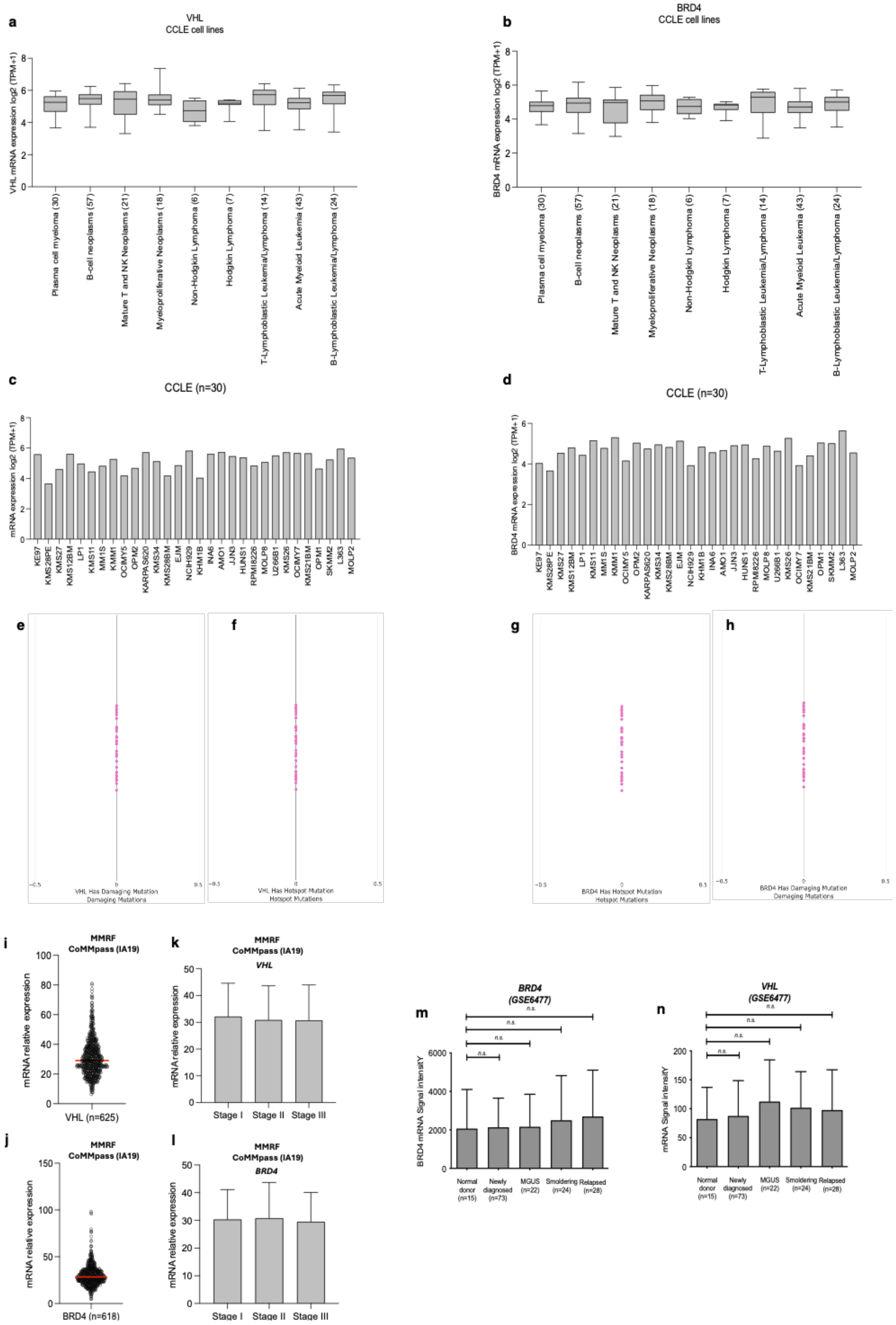
a
VHL
CCLE cell lines
VHL mRNA expression log2 (TPM+1)
Plasma cell myeloma (30)
B-cell neoplasms (57)
Mature T and NK Neoplasms (21)
Myeloproliferative Neoplasms (18)
Non-Hodgkin Lymphoma (6)
Hodgkin Lymphoma (7)
T-Lymphoblastic Leukemia/Lymphoma (14)
Acute Myeloid Leukemia (43)
B-Lymphoblastic Leukemia/Lymphoma (24)
b
BRD4
CCLE cell lines
BRD4 mRNA expression log2 (TPM+1)
c
CCLE (n=30)
mRNA expression log2 (TPM+1)
KE97
KMS28PE
KMS27
KMS12BM
LP1
KMS11
MM1S
KMM1
OCIMY5
OPM2
KARPAS620
KMS34
KMS28BM
EJM
NCIH929
KHM1B
INA6
AMO1
JJN3
HUNS1
RPMI8226
MOLP8
U266B1
KMS26
OCIMY7
KMS21BM
OPM1
SKMM2
L363
MOLP2
d
CCLE (n=30)
BRD4 mRNA expression log2 (TPM+1)
e
VHL Has Damaging Mutation
Damaging Mutations
f
VHL Has Hotspot Mutation
Hotspot Mutations
g
BRD4 Has Hotspot Mutation
Hotspot Mutations
h
BRD4 Has Damaging Mutation
Damaging Mutations
i
MMRF
CoMMpass (IA19)
mRNA relative expression
VHL (n=625)
j
MMRF
CoMMpass (IA19)
mRNA relative expression
BRD4 (n=618)
k
MMRF
CoMMpass (IA19)
VHL
mRNA relative expression
Stage I
Stage II
Stage III
l
MMRF
CoMMpass (IA19)
BRD4
mRNA relative expression
Stage I
Stage II
Stage III
m
BRD4
(GSE6477)
n.s.
BRD4 mRNA Signal intensitY
Normal donor (n=15)
Newly diagnosed (n=73)
MGUS (n=22)
Smoldering (n=24)
Relapsed (n=28)
n
VHL
(GSE6477)
n.s.
mRNA Signal intensitY
Normal donor (n=15)
Newly diagnosed (n=73)
MGUS (n=22)
Smoldering (n=24)
Relapsed (n=28)

Supplementary Figure 4. The expression and mutational analysis of VHL and BRD4 in cells and patients. (a) Overall expression of VHL and (b) BRD4 across hematological cancer cells using the CCLE database (www.depmap.org). Box plots showing mRNA expression levels in plasma cell myeloma (n=30), B-cell Neoplasms (n=57), Mature T and NK neoplasms (n=21), Myeloproliferative Neoplasms (n=18), Non-Hodgkin Lymphoma (n=6), Hodgkin Lymphoma (n=7), T-Lymphoblastic Leukemia/Lymphoma (n=14), Acute Myeloid Leukemia (n=43) and B-Lymphoblastic Leukemia/Lymphoma (n=24). (c) mRNA expression of VHL and (d) BRD4 in 30 MM cell lines available at the CCLE database. Graphpad version 9 was applied to visualize the expression data. (e-h) Lack of VHL and BRD4 hotspot or damaging mutations in MM cell lines. VHL (e,f) and BRD4 (g,h) mutational signatures in the CCLE dataset. (i) Baseline expression of VHL (n=625) and (j) BRD4 (n=618) expression in CD138+ cells derived from MM patients enrolled in the MMRF CoMMpass study (release IA19-https://research.mmrf.org). Red line represents the median value. (k, l) Expression level of VHL and BRD4 in 3 different stages of MM patients derived from MMRF CoMMpass database. VHL: n=209-I, n=222-II, n=160-III; BRD4: n=203-I, n=220-II, n=166-III). (m, n) Equal expressions of VHL (k) and BRD4 (l) in CD138+ cells derived from healthy donors (HD) as well as patients with MGUS (MGUS), SMM (SMM), newly diagnosed (ND) and relapsed/ refractory (RR) MM (GSE6477 dataset, n=162). t-test analysis was used to determine the significant difference between groups. n.s. indicates that the p value is not significant ($p \geq 0.05$). Healthy donors (HD, n=15), Monoclonal gammopathy of undetermined significance (MGUS, n=22), Smoldering multiple myeloma (SMM, n=24), Newly Diagnosed (ND, n=73), Relapsed/refractory (RR, n=28).

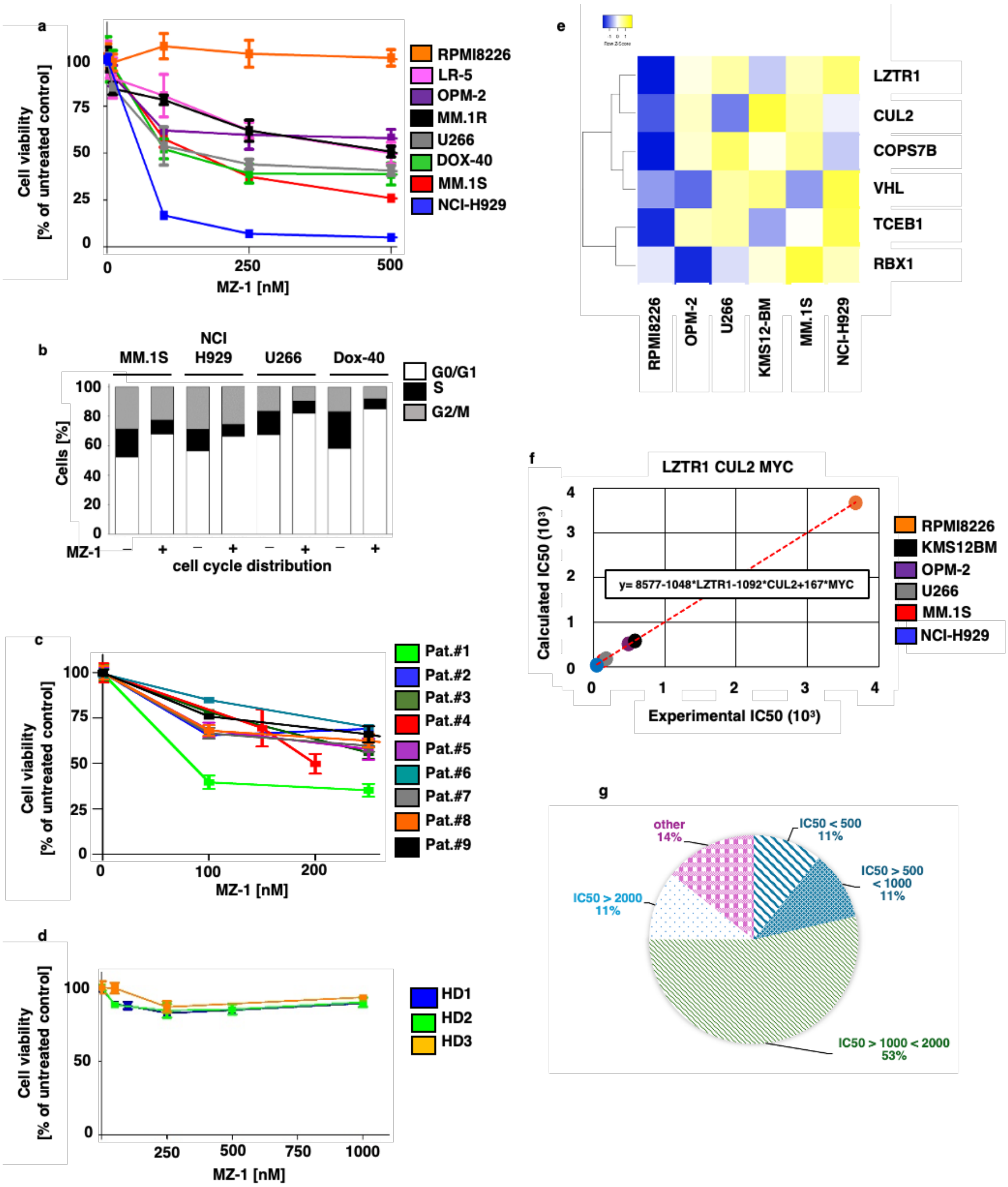

Supplemental Figure 5. Anti-MM activity of the VHL recruiting BRD4- PROTAC MZ-1. (a) MZ-1 induces inhibition of MM cell lines. Indicated MM cell line cells were treated for 24h with indicated doses of MZ-1. Cell viability was measured by MTS assay. Data in c-e represent mean ± SD for triplicate samples. (b) MZ-1- induced cell cycle arrest. Indicated cell lines were treated for 24 hours with MZ-1 (100nM) or diluent control (dimethyl sulfoxide, DMSO), stained with propidium iodide and analyzed by flow cytometry. Histograms show proportion of cells in different phases of the cell cycle. Representative of three independent experiments. (c) MZ-1 induces inhibition of patient derived MM cells but not healthy donor Peripheral Blood Mononuclear Cells (PBMCs). Primary CD138+ MM cells

isolated from 9 patients (Pat) (c); or PBMCs isolated from 3 healthy donors (HD) (d) were treated for 24h with indicated doses of MZ-1. Cell viability was measured by MTS assay. Data in c, d represent mean ± SD for triplicate samples. (e) Heatmap of mRNA expression values (log2 and Z-score scaled across genes) for in indicated MM cell line cells was obtained from data extracted from the Cancer Cell Line Encyclopedia (CCLE) database. (f) A combined index of LZTR1, CUL2, and MYC mRNA levels predicts sensitivity to MZ-1 in MM cell lines. Cell death of MM cell line cells was determined after 24 hours MZ-1 treatment by cytotoxicity assays and blotted against the multiple linear regression equation [IC50=8577-(1048×LZTR1)-(1092×CUL2)+(167×MYC)]. (g) Percentage of MM cell lines based on the calculated IC50s of MZ-1 (nM) in subgroups <500nM; >500nM < 1000nM; > 1000nM < 2000nM; > 2000nM; and other (not evaluable).
